# Patient out-of-pocket costs for guideline-recommended treatments for erectile dysfunction: a medicare cost modeling analysis

**DOI:** 10.1038/s41443-024-00903-9

**Published:** 2024-06-26

**Authors:** Vi Nguyen, Alysha M. McGovern, Sirikan Rojanasarot, Darshan P. Patel, Samir Bhattacharyya, Liesl M. Hargens, Olubiyi Aworunse, Tung-Chin Hsieh

**Affiliations:** 1 Department of Urology, UC San Diego Health, San Diego, CA USA; 2 Boston Scientific, Marlborough, MA USA

**Keywords:** Sexual dysfunction, Surgery

## Abstract

Patient out-of-pocket (OOP) cost represents an access barrier to erectile dysfunction (ED) treatment. We determined OOP cost for men with ED covered by Fee-for-Service Medicare. Coverage policies were obtained from the Medicare Coverage Database for treatments recommended by the 2018 American Urological Association (AUA) guidelines. OOP cost was retrieved from the 2023 Centers for Medicare & Medicaid Services Final Rule. OOP cost for treatments without Medicare coverage were extracted from GoodRx® or literature and inflated to 2022 dollars. Annual prescription costs were calculated using the published estimate of 52.2 yearly instances of sexual intercourse. Medicare has coverage for inflatable penile prostheses (IPP; strong recommendation), non-coverage for vacuum erection devices (VED; moderate recommendation) and phosphodiesterase type-5 inhibitors (PDE5i; strong recommendation), and no policies for intracavernosal injections (ICI; moderate recommendation), intraurethral alprostadil (IA; conditional recommendation), or low-intensity extracorporeal shock wave therapy (ESWT; conditional recommendation). Annual IA prescription is most costly ($4022), followed by ICI prescription ($3947), one ESWT course ($3445), IPP ($1600), PDE5i prescription ($696), and one VED ($213). PDE5i and IPP, both strongly recommended by AUA guidelines, are associated with lower OOP cost. Better understanding of patient financial burden may inform healthcare decision-making.

## Introduction

Erectile dysfunction (ED) is a common condition, with increasing prevalence with older age [1]. Epidemiologic studies have demonstrated the prevalence is as follows: 4.5% among men ages 40–45 years, 11.1% among men ages 50–55 years, and up to 52% among men ages 75–80 years [1]. However, only 10% of men with sexual problems seek medical attention, and consequently, up to 70% of men with ED are not treated [1, 2]. Untreated ED may result in withdrawal from sexual intimacy, psychosocial problems (i.e., poor self-esteem, depression, and anxiety), decreased work productivity, and reduced quality of life for both the men suffering from the condition and their partners [3–6].

Several reliable medical and surgical treatment options for ED are available [7]. The 2018 American Urological Association (AUA) guidelines for the management of ED state patients should be informed of all treatment options that are not contraindicated to determine the most appropriate treatment [7]. The needs and expectations of ED patients vary widely, and the treatment approach should be individualized according to patient preference.

It is imperative that effective ED treatments are accessible to patients, as this can result in a profound improvement in physical well-being, quality of life, self-esteem, relationships, self-worth, and productivity [8]. Treatment for ED is widely considered ‘medically necessary’ by healthcare insurers [8]. In the United States (US), many commercial insurers and Medicare have published coverage policies providing criteria for the medical necessity of treating ED [8]. High patient out-of-pocket (OOP) costs may cause care to be delayed or foregone and can lead to financial distress among patients [9]. However, physicians are often unaware of the price of medical treatments and services they provide and may not fully understand the associated OOP costs to their patients [10, 11]. A better understanding of the patient financial burden of ED treatment will inform healthcare decision-making. In the published literature, patient OOP costs associated with various ED treatments have not yet been evaluated. This study aimed to estimate the US Medicare patient financial burden of guideline-recommended treatment options for ED.

## Methods

### Model design, target patient population, time horizon, and comparators

A Microsoft® Excel®-based (Redmond, Washington, US) cost model was constructed to evaluate the patient OOP costs of guideline-recommended ED treatment options. An economic evaluation was performed from the patient perspective, with the target patient population being US men with moderate-to-severe ED covered by Fee-for-Service Medicare. The time horizon for the model was one year. The 2018 AUA guidelines for ED were used to identify recommended treatment options for men with ED [7]. The treatment options identified include oral phosphodiesterase type-5 inhibitors (PDE5i), intraurethral alprostadil (IA), intracavernosal injections (ICI), vacuum erection devices (VED), inflatable penile prostheses (IPP), and low-intensity extracorporeal shock wave therapy (ESWT). Since this study did not involve human participants and uses publicly available cost data, neither Institutional Review Board approval nor informed consent were obtained.

### Out-of-pocket cost inputs

The US Medicare Coverage Database was used to retrieve coverage policies for each of the ED treatment options [12]. Given Medicare has established national coverage for IPP, the patient OOP cost for IPP was assumed to equal the 2023 Centers for Medicare & Medicaid Services outpatient copayment maximum, which cannot exceed the Final Rule Medicare Part A inpatient deductible of $1600 [12]. Annual OOP costs for treatment options without positive Medicare coverage were either extracted from published literature or obtained from GoodRx® (Santa Monica, California, US) [13, 14]. Table 1 includes all references for OOP cost input calculations.

**Table 1:** AUA guideline-recommended treatment options for erectile dysfunction [7].

Treatment Option	AUA recommendation	Cost analysis		
		Methodology	Unit	Cost input source
PDE5i	Strong	Out-of-pocket cost without coverage by Medicare Part D	Annual prescription	GoodRx® (sildenafil, Viagra, tadalafil, Cialis, vardenafil, Levitra, avanafil, Stendra) [14]
IPP	Strong	Annual Medicare outpatient copayment cap for 2023	One procedure	CMS 2023 (CPT 54405) [12]
VED	Moderate	Out-of-pocket cost without Medicare coverage	One unit	DHHS 2013 and BLS 2023 [18, 19]
ICI	Moderate	Out-of-pocket cost without coverage by Medicare Part D	Annual prescription	GoodRx® (Caverject and Edex) [14]
IA	Conditional	Out-of-pocket cost without coverage by Medicare Part D	Annual prescription	GoodRx® (MUSE) [14]
ESWT	Conditional (Investigational)	Out-of-pocket cost without Medicare coverage	One treatment course^a^	Weinberger 2022 [13]

*AUA* American Urological Association, *BLS* US Bureau of Labor Statistics, *CMS* Centers for Medicare and Medicaid Services, *CPT* Current Procedural Terminology, *DHHS* US Department of Health and Human Services, *ESWT* extracorporeal shock wave therapy, *IA* intraurethral alprostadil, *ICI* intracavernosal injection, *IPP* inflatable penile prosthesis, *PDE5i* phosphodiesterase type-5 inhibitor, *VED* vacuum erection device.

^a^One treatment course = average of six sessions.

To calculate the OOP cost associated with oral PDE5i medications, the four Food and Drug Administration (FDA) approved medications currently available in the US (sildenafil, tadalafil, vardenafil, and avanafil) were included in the model. The dosage of each medication was based on a typical GoodRx® dose (25 mg sildenafil, 10 mg tadalafil, 10 mg vardenafil, and 100 mg avanafil). Patient costs for each medication (both tradename and generic) were obtained from GoodRx® in November 2022 and were calculated as an average cost across all GoodRx® pharmacies in the five US states with the greatest number of Medicare beneficiaries (California, Florida, Texas, New York, and Pennsylvania) [15]. The costs were then converted to annual costs based on an assumption of 52.2 instances of sexual intercourse per year among US men aged 57 to 72 years, as reported by Karraker et al. [16]. The proportion of brand versus generic utilization of PDE5i was obtained from a publicly available source that used GoodRx® to estimate the brand distribution and market share of these oral medications to obtain an overall weighted average estimate for 2022 ED medication OOP costs [17].

To calculate the OOP costs associated with IA and ICI, the same calculation approach as for oral PDE5i medications was taken. A weighted average of brand and generic was not utilized because IA and ICI are only available under tradenames in the US. For IA, the cost of a 125 mcg MUSE (alprostadil urethral suppository; MEDA pharmaceutics, Solna, Sweden) was obtained from GoodRx® to calculate the annual suppository cost to the patient [14]. For ICI, the average OOP costs of two cartridges of 10 mg Edex® (Endo Pharmaceuticals, Malvern, Pennsylvania, US) and one vial of 20 mcg Caverject® (Pfizer, New York, New York, US) were obtained from GoodRx® to calculate the annual cost to the patient [14].

The OOP cost for VED was obtained from the average internet price of each VED previously collected by the Department of Health and Human Services [18]. These costs were then inflated to 2022 dollar values using the US Bureau of Labor Statistics (BLS) Consumer Price Index [19]. The model assumed each patient would use only one VED in a given year.

The annual OOP cost for ESWT was calculated assuming only one treatment course is needed yearly. The treatment cost ($3338.28) was obtained from a previously published study that reported the costs of shock wave therapy for ED from eight populous cities in major metropolitan areas in the US [13]. According to this paper, the most common number of sessions per treatment course was six sessions [13]. The authors utilized a “secret shopper” method in order to contact clinics via telephone in order to identify cost and average number of sessions. This cost was then inflated to 2022 dollars using the US BLS Consumer Price Index [19].

To understand the potential US healthcare system economic implications, this model estimated the total OOP cost that men with ED covered by Fee-for-Service Medicare spend each year for each treatment option and reported the sum at one year and five years. This calculation referenced the estimated number of men age 65 and older with moderate-to-severe ED covered by Medicare (*n* = 254,650) previously reported in the literature [20]. This was a conservative approach, as this study specifically assessed men who may be candidates for IPP. Thus, this approach does not account for men with milder ED who may not be surgical candidates but who may still utilize more conservative treatment options. This projection assumed the cohort of men experiencing moderate-to-severe ED underwent the six distinct treatment pathways delineated in this study exclusively.

## Results

### Guideline-recommended ED treatment options and coverage policies

The 2018 AUA guidelines recommend five treatment options for men with ED: (1) oral PDE5i medications with a strong recommendation; (2) VED with a moderate recommendation; (3) IA with a conditional recommendation; (4) ICI with a moderate recommendation; and (5) IPP with a strong recommendation [7]. ESWT is considered investigational by the AUA, with a conditional recommendation [7]. Medicare has established national coverage for IPP, non-coverage for VED and PDE5i, and no published coverage policies for ICI, IA, or ESWT (Table 1) [8, 12].

### Medicare patient out-of-pocket costs for ED treatments

**Fig. 1:**
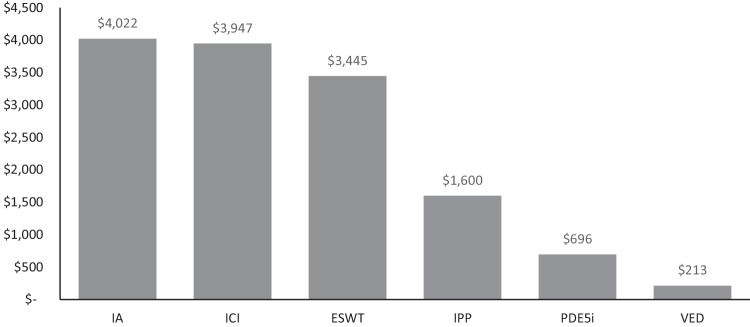
Annual patient out-of-pocket costs for guideline-recommended erectile dysfunction treatments. ESWT, extracorporeal shock wave therapy; IA, intraurethral alprostadil; ICI, intracavernosal injection; IPP, inflatable penile prosthesis; PDE5i, phosphodiesterase type-5 inhibitor; VED, vacuum erection device. Note: IPP and VED are one-time costs and do not incur repeat annual expense to patients.

An annual IA prescription was associated with the highest patient OOP costs ($4022), followed by an annual ICI prescription ($3947), one ESWT treatment course ($3445), IPP as an outpatient procedure ($1600), one year of PDE5i medications ($696), and one VED unit ($213; Fig. 1).

**Fig. 2:**
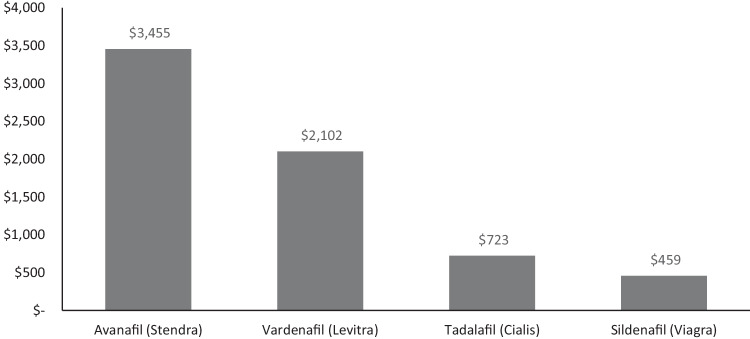
Annual patient out-of-pocket costs for PDE5i medications. PDE5i, phosphodiesterase type-5 inhibitor.

Among PDE5i medications, avanafil is associated with the highest annual patient OOP costs ($3455), followed by vardenafil ($2102), tadalafil ($723), and sildenafil ($459; Fig. 2). This figure includes costs derived from a combination of brand name and generic formulation for each medication. A breakdown of annual costs associated with brand name versus generic for each of these medications is included in Table 2.

**Table 2:** Annual patient out-of-pocket costs for brand name versus generic PDE5i medications.

PDE5i medication	Brand name annual patient cost	Generic annual patient cost
Avanafil (Stendra)	$3455	Generic not available
Vardenafil (Levitra)	$2788	$605
Tadalafil (Cialis)	$3720	$186
Sildenafil (Viagra)	$3091	$147

*PDE5i* phosphodiesterase type-5 inhibitor.

**Fig. 3:**
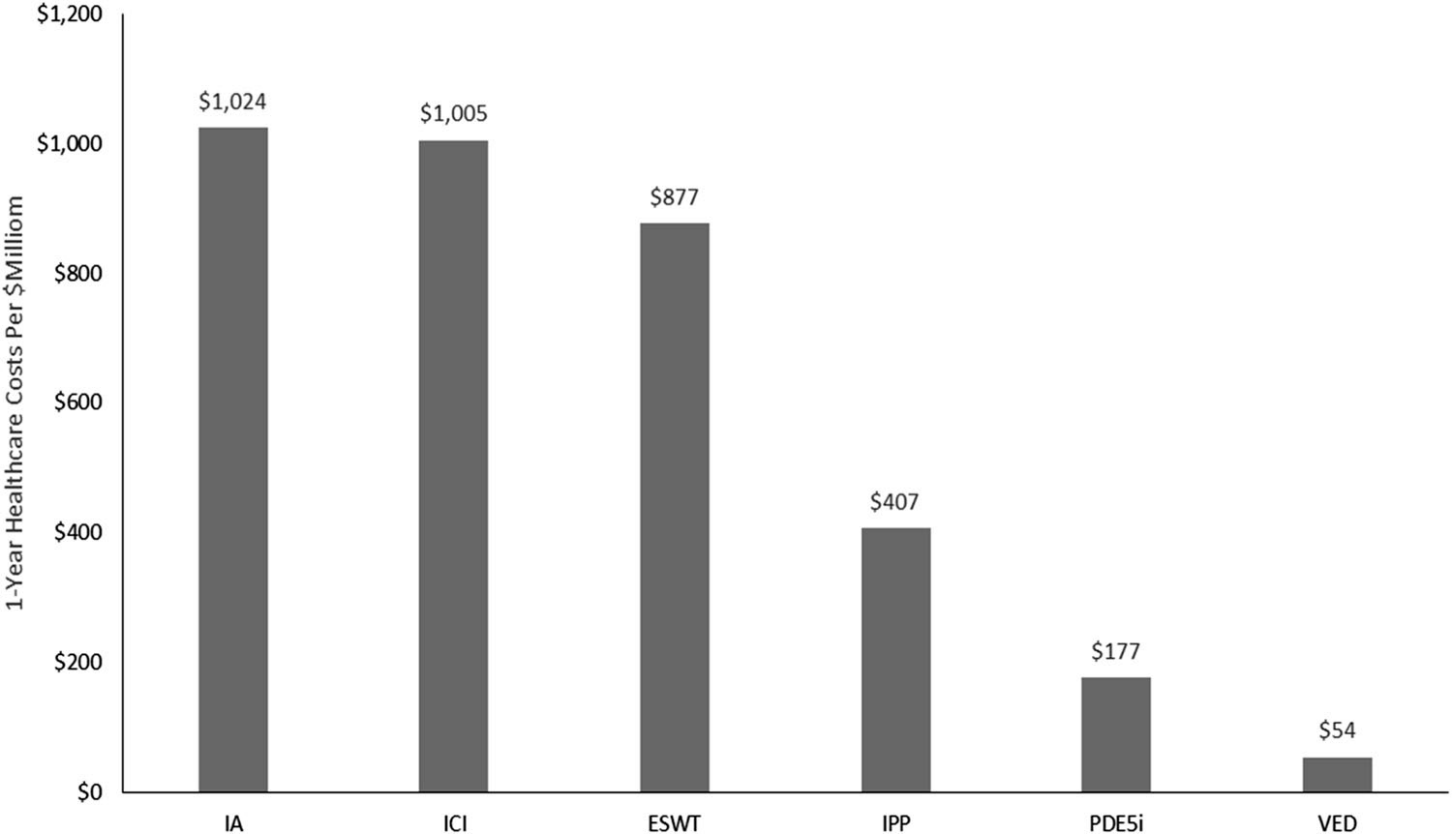
1-year projected cumulative national healthcare out-of-pocket costs of guideline-recommended ED treatments among medicare fee-for-service eligible men. ED erectile dysfunction, ESWT extracorporeal shock wave therapy, IA intraurethral alprostadil, ICI intracavernosal injection, IPP inflatable penile prosthesis, PDE5i phosphodiesterase type-5 inhibitor, VED vacuum erection device. Note: IPP and VED are one-time costs and do not incur repeat, annual expense to patients.

The 1-year projections for each treatment pathway demonstrate IA is associated with the highest cumulative national healthcare OOP costs for Medicare Fee-for-Service eligible men ($1.02 billion annually), followed by ICI ($1.0 billion annually), ESWT (one time cost of $877 million), IPP (one time cost of $407 million), PDE5i ($177 million annually), and VED (one time cost of $54 million) (Fig. 3).

**Fig. 4:**
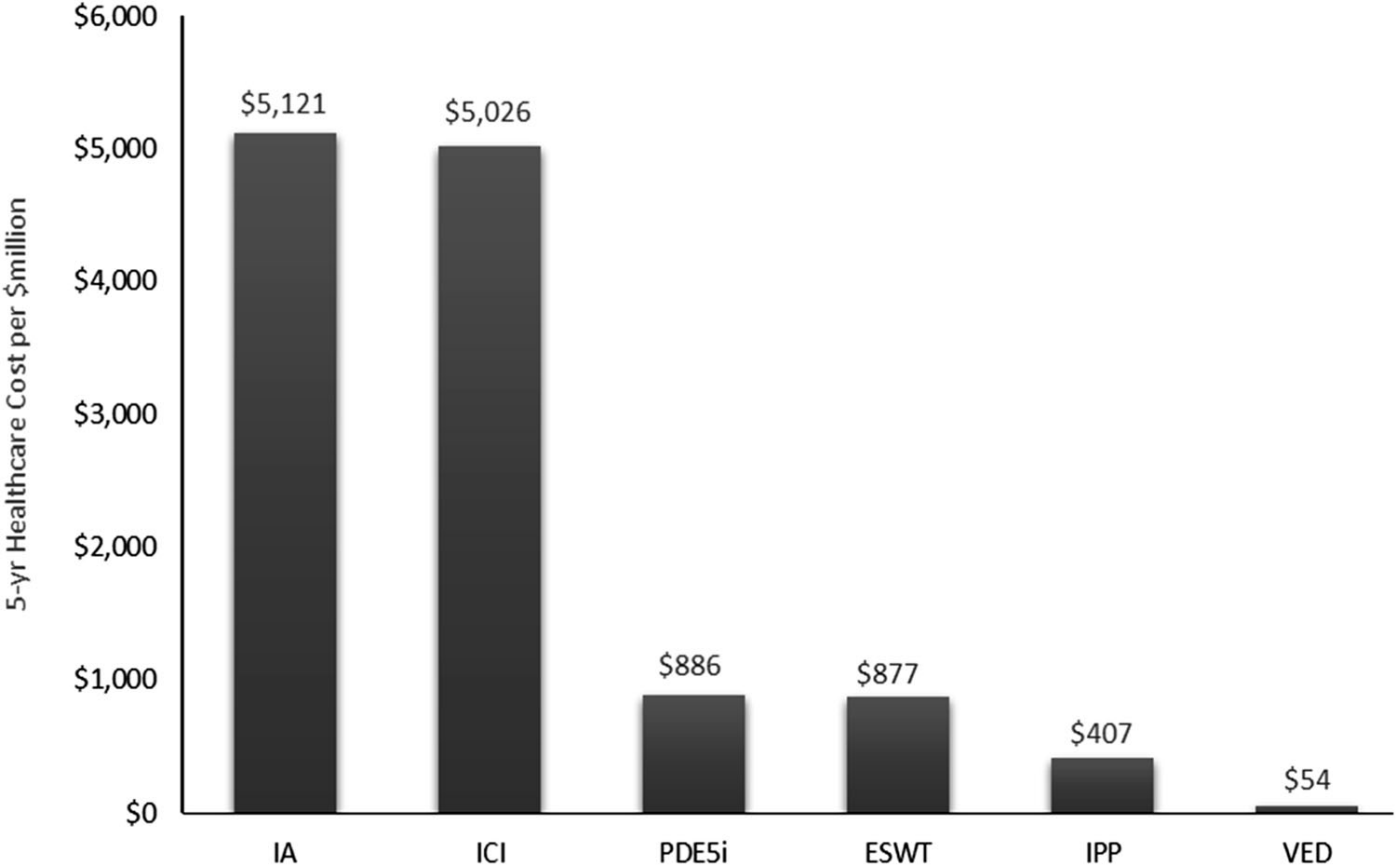
5-year projected cumulative national healthcare out-of-pocket costs of guideline-recommended ED treatments among medicare fee-for-service eligible men. ED erectile dysfunction, ESWT extracorporeal shock wave therapy, IA intraurethral alprostadil, ICI intracavernosal injection, IPP inflatable penile prosthesis, PDE5i phosphodiesterase type-5 inhibitor, VED vacuum erection device. Note: IPP and VED are one-time costs and do not incur repeat, annual expense to patients.

The 5-year projections for each treatment pathway demonstrate IA is associated with the highest cumulative national healthcare OOP costs for Medicare Fee-for-Service eligible men ($5.1 billion), followed by ICI ($5.0 billion), PDE5i ($886 million), ESWT ($877 million), IPP ($407 million), and VED ($54 million) (Fig. 4).

## Discussion

Many physicians have limited insight into the costs of, and insurance coverage for, the treatment options they recommend, resulting in underestimation of the financial burden to their patients [10, 11]. Cost awareness is important in therapeutic decision-making and cost-effective treatment [11]. High OOP costs may cause financial distress among patients and have been associated with patients delaying or foregoing necessary treatment [9]. Specifically among patients with ED, questionnaire and interview-based studies have revealed that common reasons for avoidance of treatment include cost, concern regarding side effects, as well as beliefs that the ED will spontaneous resolve, is a natural part of aging, or cannot be improved [21–23]. Given the profound impact of ED on physical well-being, quality of life, self-esteem, relationships, self-worth, and productivity, it is imperative that effective ED treatments are accessible to patients [8]. Psychological impairment associated with ED may lead to missed work days, absenteeism, and activity impairment [4]. The productivity loss burden of ED has a comparative magnitude to that of other common chronic conditions, including pain and depression [24]. Conversely, treatment for ED has been shown to be protective against the development of major depressive disorder within three years [25].

The results of this study demonstrate PDE5i medications and IPP are favorable and cost-competitive treatment options for men with ED. Both treatment options have strong guideline recommendations by the AUA and are associated with lower patient costs than IA, ICI, or ESWT (which is considered investigational).

The efficacy and tolerability of PDE5i medications for ED has been demonstrated across patients with varying etiologies and across a broad range of ED severities and ages [26]. However, approximately half of patients prescribed PDE5i discontinue therapy within one year, with the most common reasons including lack of efficacy (27%), cost (20%), and adverse effects (13%) [27]. Hence, medical therapy is inadequate for a large proportion of patients with ED.

IPP is a well-known alternative treatment option for men with ED and is associated with high patient satisfaction rates [28, 29]. A previous publication demonstrated patients with ED who underwent penile implant surgery had significantly better erectile function and treatment satisfaction rates than patients who received PDE5i and ICI at mean follow up of 19.54 months [30]. It is estimated that approximately 1.7 million men in the US who have tried and failed other treatment options for ED who could benefit from IPP [20]. IPP is designed to provide durable treatment for ED, restoring sexual function for a median device survival time of approximately 20 years without additional treatment costs [31]. Given this durability, IPP may present a less costly alternatively with a potential single lifetime occurrence as compared to recurrent costs from other therapeutic modalities. For example, utilizing the aforementioned 20 year durability, it can be extrapolated that that a one time IPP is less costly ($1600) compared to 20 years of PDE5i ($696 × 20 = $13,920). However, given it is a more invasive approach, it is critical to counsel patients regarding the potential complications, including mechanical failure, infection, shortening of penile length, change in penile sensation, or injury to local structures [32].

Medicare and many US commercial insurers have published coverage policies detailing the medical necessity of ED treatment [8]. However, only 23% of employed men who have been diagnosed with ED receive treatment paid by their employer-sponsored health plan [33]. Over the past 10 years, the rate of ED treatment with PDE5i has remained consistent; however, the use of other treatments, including IPP, VED, and ICI, has declined [33]. OOP costs may be a significant reason for the undertreatment of patients with ED, and patient financial burden may restrict patients’ ability to access optimal ED treatment. Patient financial burden may make it difficult for healthcare providers to implement ED treatment guidelines appropriately and to provide medically necessary treatment for patients with ED [8]. Furthermore, there may be disparities for some patients due to variations in medical coverage and ability to pay for treatments OOP [8]. In their call to action, Burnett et al. addressed this issue and acknowledged that while there are federal and state mandates to ensure access to treatment for women’s breast health, female-factor infertility, and gender affirmation, similar mandates are lacking in the realm of men’s sexual and reproductive health [8]. Advocacy and policy interventions may assist in reducing this disparity and improving access to care.

There are several limitations of this study, many of which are inherent to all modeling studies. Models represent a simplification of disease and treatment pathways and combine data inputs from multiple sources. Model inputs from the published literature may be out-of-date given the evolving and continued aging population dynamics, changes to clinical care, and technological innovation; however, the cost estimates obtained for this study from the literature are considered conservative. In addition, the model was structured to demonstrate OOP costs to US Medicare Fee-for-Service patients receiving one ED treatment over the course of one year. Thus, the results from this analysis may not accurately represent the OOP costs incurred by patients who try multiple ED treatment options in a given year, potentially underestimating the actual financial burden to patients. Furthermore, our model did not account for potentially lower costs associated with obtaining these medications online, particularly with the emergence of direct-to-consumer men’s health and online prescriptions. A study aimed at characterizing this model revealed that 65% offer ED treatment, yet only 10.3% had a urologist as a primary provider [34]. Given the novelty of these services, further investigation is needed to understand how this model may increase accessibility to ED treatment. Finally, the results of this modeling evaluation reflect US patients with Medicare Fee-for-Service insurance, and results may not be generalizable to patients without health insurance, patients with Medicare Advantage, Veterans Affairs, or Tricare health insurance, or patients for which clinical practice and reimbursement structure, health care accessibility, and treatment accessibility may differ.

## Conclusions

This cost model analysis estimated US Medicare patient and healthcare system OOP costs for guideline-recommended treatments for ED. IA is conditionally recommended by the AUA and is associated with the highest patient OOP costs. VED is associated with the lowest patient OOP costs but is only given a moderate guideline recommendation by the AUA. PDE5i and IPP were associated with lower patient costs than IA, ICI, and ESWT. Given their strong AUA guideline recommendations, PDE5i and IPP were found to be favorable and cost-competitive treatment options for men with ED.

## Data Availability

The datasets generated during and/or analysed during the current study are available from the corresponding author on reasonable request.
